# 

**Published:** 1900-04-14

**Authors:** 


					THE SOBPITAL. " The standard of highest purity,"?Lancet, pw  April 14th, 1900.
CADBURY'S U Tps CADBURY'S
GOOOA Vk ^ m MO COCOA
E.Y rau. NO OKU08 OR alkalik* utxo.
No. 70y. Vol. XXYIII. CSfi5E?] Saturday,
April 14, 1900.
[Subscription TBnnB,] pKICE TWOPENCE,

				

